# Bacteria Single-Cell and Photosensitizer Interaction Revealed by Quantitative Phase Imaging

**DOI:** 10.3390/ijms22105068

**Published:** 2021-05-11

**Authors:** Igor Buzalewicz, Agnieszka Ulatowska-Jarża, Aleksandra Kaczorowska, Marlena Gąsior-Głogowska, Halina Podbielska, Magdalena Karwańska, Alina Wieliczko, Anna K. Matczuk, Katarzyna Kowal, Marta Kopaczyńska

**Affiliations:** 1 Department of Biomedical Engineering, Faculty of Fundamental Problems of Technology, Wrocław University of Science and Technology, 27 Wybrzeże S. Wyspiańskiego St., 50-370 Wrocław, Poland; agnieszka.ulatowska-jarza@pwr.edu.pl (A.U.-J.); aleksandra.kaczorowska@pwr.edu.pl (A.K.); marlena.gasior-glogowska@pwr.edu.pl (M.G.-G.); halina.podbielska@pwr.edu.pl (H.P.); marta.kopczynska@pwr.edu.pl (M.K.); 2 Department of Epizootiology and Veterinary Administration with Clinic of Infectious Diseases, Wrocław University of Environmental and Life Sciences, 45 Grunwaldzki Square, 50-366 Wrocław, Poland; magdalena.karwanska@upwr.edu.pl (M.K.); alina.wieliczko@upwr.edu.pl (A.W.); 3 Department of Pathology, Division of Microbiology, Faculty of Veterinary Medicine, Wrocław University of Environmental and Life Sciences, 31 C.K. Norwida St., 51-375 Wrocław, Poland; anna.matczuk@upwr.edu.pl; 4 Independent Researcher, Leeds 0113, UK; katarzyna.m.kowal@gmail.com

**Keywords:** digital holographic tomography, photodynamic inactivation, single-cell bacteria

## Abstract

Quantifying changes in bacteria cells in the presence of antibacterial treatment is one of the main challenges facing contemporary medicine; it is a challenge that is relevant for tackling issues pertaining to bacterial biofilm formation that substantially decreases susceptibility to biocidal agents. Three-dimensional label-free imaging and quantitative analysis of bacteria–photosensitizer interactions, crucial for antimicrobial photodynamic therapy, is still limited due to the use of conventional imaging techniques. We present a new method for investigating the alterations in living cells and quantitatively analyzing the process of bacteria photodynamic inactivation. Digital holographic tomography (DHT) was used for in situ examination of the response of *Escherichia coli* and *Staphylococcus aureus* to the accumulation of the photosensitizers immobilized in the copolymer revealed by the changes in the 3D refractive index distributions of single cells. Obtained results were confirmed by confocal microscopy and statistical analysis. We demonstrated that DHT enables real-time characterization of the subcellular structures, the biophysical processes, and the induced local changes of the intracellular density in a label-free manner and at sub-micrometer spatial resolution.

## 1. Introduction

The vast majority of contemporary therapeutic procedures require use of artificial devices or implants, such as percutaneous/transdermal catheters or cannulas, percutaneous drainage, bone or tooth implants, urinary or cardiovascular stents, or cardiac valves. Such devices can be colonized by bacteria that are able to form difficult to combat biofilms and therefore create a biologically critical interface issue. The formation of microbial biofilms, i.e., on catheters, increases the risk of occurrence of bloodstream infections [1]. According to the NIH (National Institutes of Health, USA) and other reports, biofilms are involved in more than 80% of chronic inflammatory and infectious diseases, including ear infections, gastrointestinal ulcers, urinary tract infections, or pulmonary infections in cystic fibrosis patients [2,3]. Biofilms are difficult to eradicate with common antimicrobial agents because they can be nearly 1500-fold more resistant to antibiotics than planktonic, free-floating cells, and so the concept of biofilm-related diseases has been recently introduced in medical field [4,5]. The COVID-19 pandemic caused by the Severe Acute Respiratory Syndrome Coronavirus 2 (SARS-CoV-2) has shown that bacteria-related chronic inflammatory and infectious diseases (particularly pulmonary infections) can be responsible for higher mortality [6]. It was reported that during the SARS-CoV-2 pandemic, 50% of the patients with COVID-19 who died had secondary bacterial infections [7]. The formation of biofilms on the inner and outer surfaces of the tracheal cannula, which increases the risk of ventilator-associated pneumonia, was reported [8,9]. The most common infections are caused by opportunistic pathogens, considered non-pathogenic, such as *Staphylococcus* spp., which are part of the resident flora. 

Bacterial colonization by *S. aureus* on external bone fixators pins [10,11] as well as contamination of wounds by *E. coli* biofilms [12] have been reported. Bacterial biofilm formation is a multistep process in which microorganisms attach to and grow on a surface with the production of an extracellular matrix, mainly polysaccharides and proteins. Biofilm formation takes place in three stages: attachment of bacteria to a surface, maturation, and dispersion-shedding of parts of the biofilm into the aqueous medium [13]. In this study, two biofilm-forming bacterial species were used: *E. coli* and *S. aureus*. Within 2 h, *S. aureus* could attach to the surface, which proceeded with biofilm gene expression [14]. Within 6–8 h, the biofilm completed initial proliferation and entered the maturation stage [15]. Similar times were observed for *E. coli* biofilm formation, with mature biofilm typically formed within 24 h [16].

So far, the most effective method of eliminating a potential bacterial infection revealed to have formed biofilm on a medical device has been its removal, followed by systematic and/or local antibiotics therapy [17]. Additionally, a variety of topical skin disinfectants can be applied to avoid transcutaneous contamination [18]. In some studies, a combination of at least two antimicrobial agents was found to be effective in reducing the incidence of pin-tract infection, e.g., chlorhexidine and silver sulphadiazine [19]. Studies have shown that, e.g., polyhexamethylene biguanide is effective against a diverse range of bacteria, including *Staphylococcus aureus*, methicillin-resistant *S. aureus, S. epidermidis*, *P. aeruginosa*, *E. coli*, and *K. pneumoniae*, which are common microorganisms present on infected pins [20]. However, because one of the side effects of polyhexamethylene biguanide is a contact allergy, it is therefore often listed as a contraindication in patients at high risk [21]. The majority of antibacterial agents are available in aqueous form and are applied/administered by spraying or soaking; they remain in the target area for a very short time after application. Therefore, novel antimicrobial materials with long-term activity is a potential route for reducing the occurrence or even completely limiting bacteria biofilm formation and wound infections. There are many wound exudate rheology modifiers available for application in wound care, but the recent trends favor multifunctional polymers, especially those with antimicrobial and antifilm-forming properties.

By providing a novel diagnostic tool and a method of antibacterial photoactive material’s characterization on single bacteria cell level by digital holographic tomography (DHT), the present study attempts to address some of the problems related to biofilm formation. This novel quantitative phase-imaging technique [22] is based on optical diffraction tomography, which enables the characterization of morphological, optical, and biochemical properties with a sub-micrometer [23,24,25] or even sub-nanometer resolution [26]. The theoretical lateral and axial resolution of the DHT systems are equal to 124 nm and 397 nm, respectively [27,28]. In this technique, the phase and amplitude of the scattered light waves are retrieved from the series of digital holograms registered at different angles of the illuminating beam and are then processed for reconstruction of the three-dimensional (3D) refractive index (RI) maps or tomograms. The variation of the local RI values is associated with the examined object morphology but also with local fluctuations of its density, dependent from the chemical composition. Digital holographic tomography is based on a limited-angle holographic tomography [22,26,29,30,31] approach, which is one of the most popular versions of optical diffraction tomography used in biomedical examination. Living cells are complex structures that contain numerous organelles with different refractive indices [25,32]. Even bacteria, being prokaryotic cells, exhibit variations of the refractive index value related to their internal structure (cell wall, cytoplasm, nucleoids) or to external factors. Moreover, DHT enables non-destructive, label-free 3D imaging with lower phototoxicity and no photobleaching.

Antimicrobial photodynamic therapy (APDT) involves the use of low-power lasers with appropriate wavelength to selectively kill microorganisms treated with a photosensitizer [33]. For the purpose of the current study, a new antibacterial photoactive material was developed and used to validate the potential of DHT application for the characterization of bacteria single-cell–photosensitizer interaction. Photosensitizers immobilized within a polymer matrix were used to obtain and utilize an antimicrobial photoactive material. As a polymer, the ammonium acryloyldimethyltaurate copolymer (AVC), being a rheology modifier widely used in pharmacology and dermatology, was applied [34,35,36]. The modifier is a copolymer that consists of acrylamido-2,2-dimethylpropanesulfonic acid and vinylpyrrolidone, with an alternating arrangement within the macromolecule. It is characterized by a high degree of cross-linking and exhibits strong interactions with polar solvents. AVC was selected as a polymer carrier for the porphyrins: pheophorbide a (Pheo) and chlorin e6 (Ce6) as well as for the photosensitizer for APDT. The AVC stabilizes the monomeric structure of porphyrins, and as a result, it enhances the photoactivity of the photosensitizer [37,38]. The antimicrobial efficacy was examined against two bacteria species: *E. coli* (Gram-negative) and *S. aureus* (Gram-positive). The photodynamic effect, resulting from irradiation of porphyrins, led to the photodynamic inactivation (PDI) of bacteria cells. The DHT was used to assess the efficiency of this process, as well as to visualize the accumulation of the photosensitizers inside the single living bacterial cells. Recently, it was reported that the DHT can be successfully used for characterization of the dynamic changes of the 3D refractive-index distributions of living bacteria associated with the changes of their morphology in response to antibiotics [28]. However, based on our knowledge, this paper reports the first attempt to use DHT in the examination of a single bacteria cell’s penetration by photosensitizers, to assess antimicrobial efficiency, and to visualize the photo-inactivated cells. Obtained results confirm the statistically significant differences of the averaged 3D-RI values, indicating the accumulation of the photosensitizers and the photo-inactivation of the single bacteria cells. Moreover, it was verified that DHT can be used for evaluation of APDT efficacy at a single bacterial cell level. Therefore, it can be applied as a non-destructive, less time-consuming, and more cost-effective alternative versus other microscopic techniques conventionally used for these purposes.

## 2. Results and Discussions

### 2.1. Photoactive Materials and Photosensitizers Characterization

**Figure 1:**
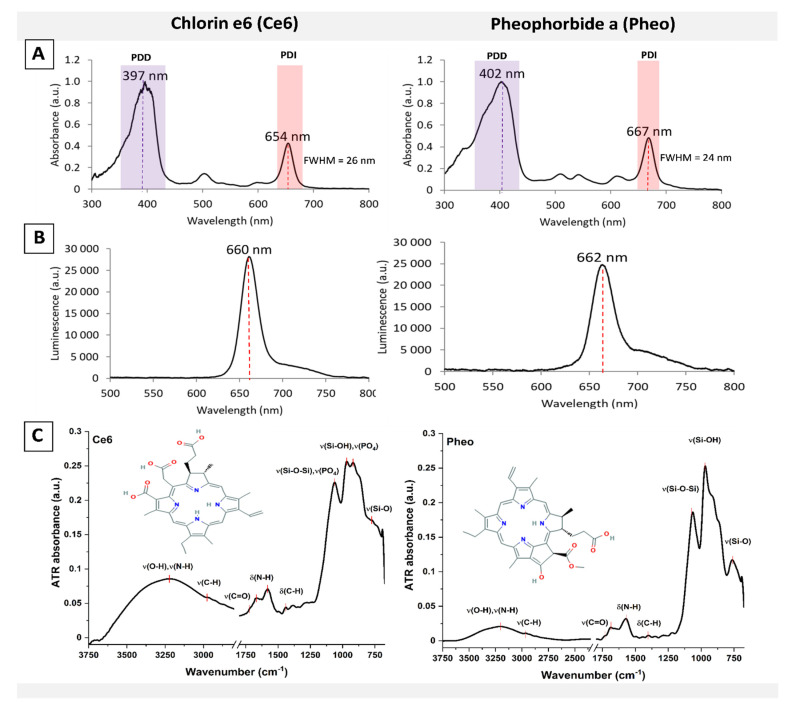
The characterization of the PS’s absorption (**A**) and luminescence spectra (**B**) for excitation at 405 nm with central wavelengths and full width at half maximum (FWHM) of spectral bands, and (**C**) attenuated total reflectance—Fourier transform infrared (ATR-FTIR) spectra of PSs in PBS buffer deposited on the silica glass surfaces.

The representative absorption and luminescence spectra of the photosensitizers (PS) are presented in the Figure 1. For each photosensitizer, the relevant spectral bands (Figure 1A) were used for photodynamic inactivation (PDI) of bacteria, and the photodynamic diagnosis (PDD) spectral bands were used to determine the efficiency of cells’ penetration by PSs.

The reference ATR-FTIR spectra of both PSs used in further experiments are shown in Figure 1C. Ce6 and Pheo exhibit the typical absorption bands of porphyrins [39,40,41]. The broad absorption band at ~3250 cm^−1^ is attributed to O-H and N-H stretching vibrations, while weak bands in the range 3000–2800 cm^−1^ relate to stretching vibrations of C-H groups.

In the range of 1750–1500 cm^−1^, bands arising from the stretching and deformation vibration of CO groups are observed, and in the range of approximately 1690–1575 cm^−1^, bands of NH groups are observed. The spectral region between 1200 and 700 cm^−1^ is dominated by glass signal due to a very thin layer of deposited dyes, as well as due to phosphate bands from PBS buffer. The wavelength (655 nm) of the laser used for photoexcitation (Figure 1B) was in the range of determined FWHM of the PDI bands. The absorption spectra (Figure 1A) of the photoactive materials (AVC-Ce6, AVC-Pheo) were analogous for PSs in the PBS buffer.

After laser irradiation (λ = 655 nm) for 2 min, significant changes in the spectrum shape were observed for both types of PSs (see Figure S1A in Supplementary Materials). However, the most evident modifications were observed for Ce6. The exposition of the Ce6 to the laser radiation caused a severe distortion of the porphyrin structure. The most noticeable is a redshift (toward lower wavenumber values, longer wavelengths) of the band arising from the bending vibrations of amine (N-H) at 1573 cm^−1^, whilst simultaneously changing in the intensity of carbonyl (C = O) signals at 1690 and 1660 cm^−1^. These bands can be assigned to the C = O stretching mode of the free and coordinated 9-keto groups [42]. 

In the case of Pheo, only a slight red shift for δ(NH) band is observed. Moreover, an analysis within the high-frequency range clearly showed that Ce6 was the more efficient source of reactive oxygen species (ROS). The maximum position of the combined water OH stretching and NH stretching band was shifted to higher wavenumber (i.e., higher energy and higher frequency) toward longer wavelengths approximately 120 cm^−1^ and 90 cm^−1^ for Ce6 and Pheo, respectively. This blue shift is caused by hydrogen bonds breaking and the formation of ROS, such as hydroxyl radicals [43,44].

Although ATR-FTIR spectra of AVC material are dominated by bands of ammonium acryloyldimethyltaurate/vinylpyrrolidone copolymer (see Figure S1B in Supplementary Materials), some spectral features allow the assumption that PS’s molecules are attached to the surface. The subtle broadening of δ(NH) and ν(CO) bands in the range of 1800–1500 cm^−1^, as well as the presence of additional bands at ~1740 and 1400 cm^−1^, corresponding to ester C = O and C-C aromatic groups, are detected. The position of the C = O stretching band in both AVC–PS spectra is located at higher wavenumbers than in the corresponding spectra of non-bound PSs and appears at the typical ester range. As expected, the irradiation process did not affect the AVC (see Figure S2 in Supplementary Materials).

### 2.2. Qualitative and Quantitative Analysis of the Single Cells’ RI Variations Caused by Photosensitizer Penetration

**Figure 2:**
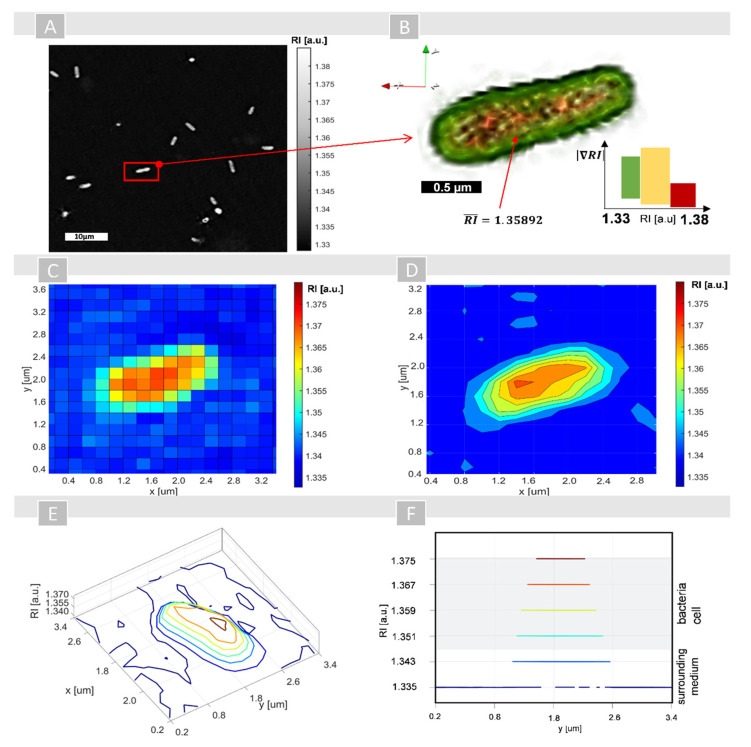
The exemplary results for *E. coli* cell on AVC material (non-photoactive): (**A**) reconstructed 2D-RI map (raw-data) with *E. coli* cells on the surface of AVC material; (**B**) 3D rendered and digitally stained single cell; (**C**) averaged 3D-RI distribution of single cell from all 2D-RI maps (slices) on which the cells were present; (**D**) distribution of averaged 3D-RI isolines; (**E**) pseudo-3D representation of averaged RI distribution; (**F**) cross-section of the isolines for different averaged 3D-RI values corresponding to the region occupied by a single cell.

The RI value is closely related to the mass concentration in biological samples [45]. According to the proposed approach, the penetration of cells by PSs is accompanied by a change of the cell’s density, which leads to the increase of the single cell’s RI values. To confirm this assumption, an analysis of the variation of average RI values of the single cells incubated on AVC material and photoactive material (AVC-Ce6, AVC-Pheo) was performed. The exemplary results for *E. coli* cells grown on AVC material and the representation of RI data processing are shown in Figure 2.

The numerically reconstructed 3D-RI distributions contained a series of 2D-RI maps (see Figure 2A), which were used for 3D rendering and digital staining of the cells based on their RI values (see Figure 2B). Green color corresponds to the outer regions of bacteria cells (cell wall and membranes), while yellow and red colors correspond to the cell interior (cytoplasm, nucleoids, ribosomes, etc.). After segmentation, it is possible to extract the region occupied by the cell in all slices of the 3D-RI distribution by selecting the voxels that have RI higher than a specific value corresponding to the RI of the medium. Then, the averaged RI values were determined by averaging RI values from pixels of the cells’ region from each 2D-RI map (slice), which enabled the 2D representation of the 3D-RI distribution of single cells (Figure 2C). However, due to the limited sampling conditions (wavelength of the used light source and resolution of the used detector) affecting the analysis of the intracellular spatial variations of the RI values, the additional processing of RI data had to be applied. The use of isolines corresponding to the planes of equal values of the RI inside the cell (see Figure 2D,E) were determined and enabled a more precise examination of the RI changes, revealing local variations of the intracellular density. Such representation of the RI data demonstrates that digital staining based on the local changes of RI values enables the direct localization of single cells and distinguishes the region occupied by a single cell from the surrounding medium (see Figure 2F). 

A significant variation of the RI values from 1.3350 (corresponding to NaCl RI) to 1.3390 of the surrounding medium was documented and related to the interaction of AVC material with NaCl, leading to the local density variation. The additional measurement of the RI of AVC material with NaCl solution on the Abbe refractometer confirmed this observation, where the averaged RI was equal to 1.3371. Based on the performed analysis, it was possible to determine the range of RI values’ variation inside the region occupied by a single cell: 1.34721–1.37891 for *E. coli* and 1.3512–1.38921 for *S. aureus*. The results obtained for *E. coli* correspond to the results from the alternative DHT technique, which were reported recently [28]. The averaged 3D-RI of *E. coli* cells, in this case, was equal to 1.35892, and the standard deviation was equal to 0.00189, while for *S. aureus* these values were 1.34988 and 0.00101, respectively.

However, the direct estimation of the border value of RI indicating the boundaries between the surrounding medium and cell is a subject of considerable uncertainty. The outer cell’s structures include not only the cell envelope consisting of cell wall (20–80 nm thick for Gram-positive and 1.5–10 nm for Gram-negative) and plasma membrane (about 7.5 nm thick) but also flagella and pili (or fimbriae) responsible for bacteria motility, adhesion, uptake, and excretion of proteins and DNA [46,47,48]. Structurally, bacterial flagella are long structures (3 to 12 µm), with a diameter in the range from 12 to 30 nm, while pili are 0.3–1.0 μm in length and about 7 nm in diameter. Therefore, the size of these structures (thickness, diameter) is below the lateral resolution of the DHT, and it is not possible to distinguish them from the medium. In consequence, the RI value near the cell will be subject to local variation caused by the random locations of these structures in the medium surrounding the cell and local changes of the density, which limit the determination of the boundary value of the RI that allows the region of the cell and external medium to be directly distinguished. The RI of these regions can be treated as an averaged value of the higher RI flagella/pili and lower RI of the surrounding medium. Moreover, the outer regions of bacterial cells consist of cell walls, membranes, or capsules that exhibit some permeability necessary for uptake of dissolved nutrients. The plasma membrane enables the transport of the nutrients based on facilitated diffusion, active transport, and group translocation, which can lead to local changes of the density of peripheral regions of the cell during the opposite transport of substrates to the concentration gradient from the external environment (external medium with lower RI) to the cellular interior with higher RI. In our opinion, the above reasons may explain the lower RI values in the peripheral region of the bacteria cells.

Moreover, in the context of the determined averaged RI of bacteria cells, it should be pointed out that these values correspond to the average RI of a single cell and variation of the RI values inside the single cell, however not to the averaged RI of multiple cells suspension, as mainly reported in previous studies based on immersion refractometry [49,50]. Nonetheless, as it will be shown in Section 2.4, during the reproduction process the RI distribution of single cells is significantly changing. Therefore, the determined RI of the cell’s suspension will never be an averaged value of all cells with the same RI distribution because cells at different stages of cell development will be present in the same suspension. There were also some reports on determination of the RI of single bacteria cell by optofluidic immersion refractometry [51], where the averaged RI of a single cell was determined by appropriately matching the RI of the media in which the cells were suspended. However, the RI values of the subcellular structures such as cytoplasm and nucleoid [50] are changing depending on the kind of medium used [32]. Additionally, the already reported results of using optical diffraction tomography to reconstruct the 3D-RI distributions of the microalgae [24], eukaryotic cells [52], and their internal structures [32]—chromosomes, cytosol, cell membrane—confirm our concerns because, depending on the type of nutrient medium, osmolality, temperature, or fixation procedures [53], significant variation of the RI values was observed. Therefore, the use of the DHT or quantitative phase imaging for characterization of single bacteria cells requires standardization and strict control of the cell’s culturing and measurement conditions to obtain comparable and repeatable results.

**Figure 3:**
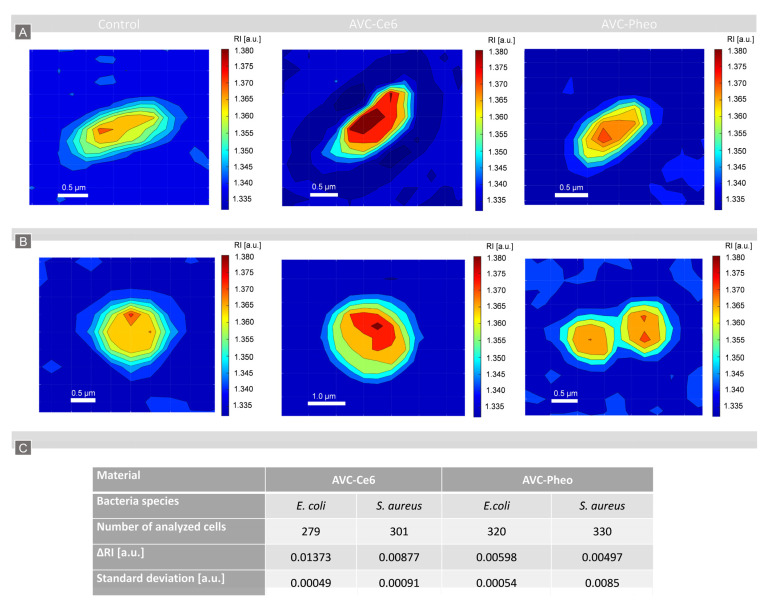
The representative 2D-RI maps of single *E. coli* (**A**) and *S. aureus* (**B**) cells on AVC (control), AVC-Ce6, AVC-Pheo materials and the averaged RI differences with standard deviations (**C**).

For the determination of the bacteria cells’ penetration by examined photosensitizers (Ce6, Pheo) the dark-controls of AVC-Ce6/AVC-Pheo materials and only AVC material were used to define the change of the refractive index of the single cells caused by the accumulation of the photosensitizers. This change was estimated by the difference between the averaged refractive index of all cells incubated on AVC material (reference) and photoactive materials (AVC-Ce6/AVC-Pheo). Results are shown in Figure 3 and Figure S3 in Supplementary Materials. 

For all examined bacteria species and the photoactive materials, an increase of the averaged RI of single cells was observed. In case of the AVC-Ce6 material, more substantial changes of the RI were noted for *E. coli* cells than for *S. aureus*. Moreover, a greater difference of the averaged RI values was observed for these two bacteria species in the case of AVC-Ce6 material than in AVC-Pheo. Obtained results indicate considerably more change in averaged RI of single cells for AVC-C6 material.

In our approach, we assumed that the greater change of the averaged RI of cells indicated a more effective accumulation of the photosensitizer inside the bacteria cell. To determine whether the observed changes of the averaged RI of cells caused by PS penetration were statistically significant, a one-way ANOVA was performed. To obtain a representative set of data for this analysis, at least 279 cells of each species were examined. The normality assumption of the average RI values of single cells was confirmed by the Anderson–Darling test at 5% significance level. The ANOVA results are depicted in Table S1 in Supplementary Materials. The estimated *p*-value for the *F*-statistic is significantly smaller (6.159 ×10^−32^) than the significance level (0.05), which means that the test rejected the null hypothesis that all group means were equal. It indicates that there exists a statistically significant difference between the analyzed groups corresponding to the averaged RI values of bacteria cells (*E. coli, S. aureus*) on AVC, AVC-Ce6, and AVC-Pheo materials. Moreover, the variability between groups was higher than the variability within the groups. Obtained results of the ANOVA indicate that the presence of the photosensitizers in the AVC material significantly influenced the averaged RI value of the single cells. Therefore, the performed examination indicated that the analysis of the 3D-RI distribution of single bacterial cell could be used for characterization of the sub-micrometer local changes of the intercellular density associated with the accumulation of the photosensitizers inside the single cells. 

**Figure 4:**
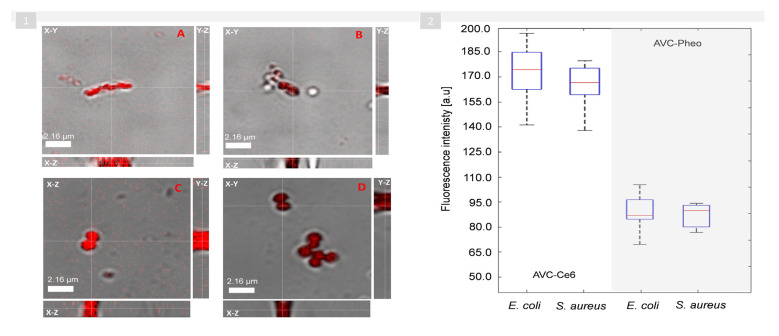
(**1**) Representative confocal 2D microscopic images (combined DIC-fluorescence images) of *E. coli* cells penetrated by Ce6 (**A**), Pheo (**B**), and *S. aureus* cells penetrated by Ce6 (**C**) and Pheo (**D**) photosensitizers; (white-dashed lines indicate the planes for which the axial (X-Z, Y-Z) cross-sections (∆z = 3µm, with resolution: 0.04 µm) were extracted). (**2**) The boxplot representing the variation of the fluorescence intensity in the region of single cells.

To confirm that the change of the averaged RI of the bacteria cells incubated with photoactive material is related to the effectiveness of the cell’s penetration by the photosensitizer, an additional examination was performed by means of scanning fluorescence confocal microscopy. The *E. coli* and *S. aureus* cells incubated for 8 h with the photosensitizer were examined to prove whether photosensitizers were accumulating in the cell’s wall or inside of the cell. After photoexcitation of the photosensitizers by the laser light with a wavelength equal to 405 nm (corresponding to the PDD absorption bands of both photosensitizers), fluorescence images of single cells, were registered (see Figure 4). 

The analysis of the differential interference contrast (DIC) and fluorescence images of cells as well as axial cross-sections (X-Z, Y-Z) resulted in the observation that the fluorescence signal is spatially overlapping all-regions occupied by the bacterial cells—not only in the cells’ capsule, cell wall, or cytoplasm membrane, but also inside of the cells of both bacteria species (Gram-positive and Gram-negative). The results indicate the presence of photosensitizers inside the cells, which confirms the ability of the used photosensitizers to penetrate through the bacterial cell’s wall into the cell of both examined species (Gram-positives and Gram-negatives). Anionic PSs such as Ce6 are not taken up into bacterial cells via simple diffusion. Uptake of anionic PS may be mediated through a combination of electrostatic charge interaction and by protein transporters, and Pheo (cationic PS) transport is mediated by electrostatic interactions and self-promoted uptake pathways. This pathway involves the binding of the cationic molecules to the outer membrane, which is rich in lipopolysaccharides (LPS), resulting in the progressive displacement of divalent cations (both Ca^2+^ and Mg^2+^ stabilize the LPS structure via electrostatic bonds), thereby weakening the outer membrane. The destabilization of the LPS layer results in the progressive weakness of the permeability barrier. The presence of cationic dye in the AVC material results in the widening of the crack in the LPS layer [54]. Efflux pumps are bacterial transport proteins that are involved in extrusion of substrates from the cellular interior to the external environment. Pheo is a natural product identified as an efflux pump inhibitor (EPI) [55,56].

It was proven that it is possible to photodynamically inactivate Gram-negative bacteria without photosensitizer accumulation in the bacterial cells. This fact is especially interesting, considering that the development of resistance may be prevented, leaving the active components (PS) outside the bacterium [57]. However, the fluorescence intensity of the obtained images indicates that the penetration of both bacteria species cells is more effective in case of the Ce6 than Pheo photosensitizer. Moreover, the comparison of the fluorescence signal intensity (see Figure 4(1)) revealed that the concentration of the Pheo photosensitizer inside the cells is significantly lower than in case of theCe6, which indicates the significantly lower penetration of the cell by this photosensitizer. These observations were also confirmed by the analysis of the discrete fluorescence intensity of single *E. coli* and *S. aureus* cells (see Figure 4(2)), demonstrating the higher fluorescence intensity in the case of the AVC-Ce6 material, which corresponds to the accumulation and concentration of the photosensitizers inside the cells. These results are consistent across the changes in the average RI of examined cells because, for AVC-Pheo material, the fluorescence intensity was significantly lower than for AVC-Ce6 material (see Figure 3). Therefore, the high correlation of the changes of the RI value of the cells, along with the efficiency of the cells’ penetration by examining photosensitizers, proved the capability of digital holotomography in the characterization of local density changes in single cells. Moreover, these results indicate that during the excitation of accumulated photosensitizer by light with a wavelength of 655 nm used to get the photodynamic inactivation, the generated free radicals destroy not only the bacterial membranes (cell wall, cytoplasm membrane) but also most probably the nucleoids present inside the cell.

### 2.3. Photodynamic Inactivation of Bacteria Cells Revealed by DHT and Its Quantitative Analysis

**Figure 5:**
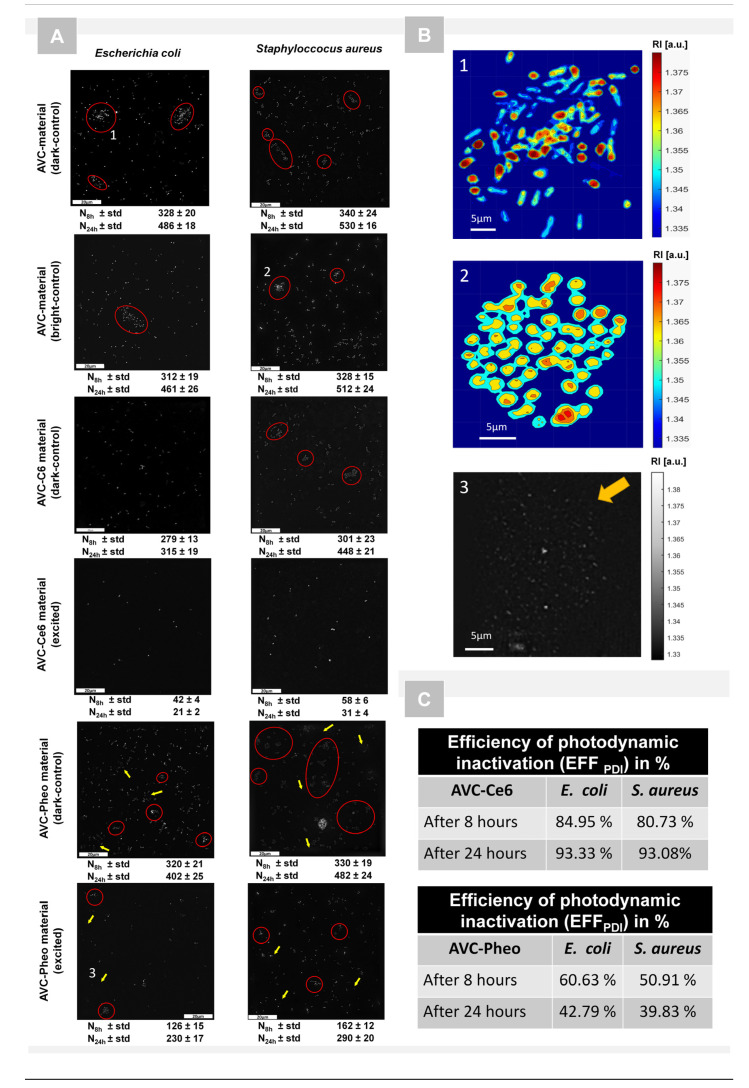
(**A**) Representative 2D-RI maps of *E. coli* and *S. aureus* cells grown on the surface of three kinds of AVC materials (24 h after photodynamic treatment) mean values of the cells’ number (N_8h_, N_24h_) and its standard deviation 8 and 24 h after photoexcitation. (Red ellipses indicate the bacterial cells clusters characteristic for biofilm formation; yellow arrows indicate the photosensitizer’s aggregates in the material). (**B**) The exemplary enlarged 2D-RI maps of characteristic structures (cells clusters—(**1**,**2**); aggregates—(**3**)) indicated on (**A**). (**C**) The efficiency of the photodynamic inactivation *EFF_PDI_* (detailed description in Section 4.8).

The holotomographic technique was used to record a series of digital holograms and reconstruct the 3D-RI maps. As it was confirmed in the previous section, the bacterial cells have different averaged RI values due to interaction with different photoactive materials, which makes this method suitable for bacteria cells visualization and characterization of cell penetration. In this section, the results of the examination of the induced photodynamic inactivation of bacterial cells are presented and based on RI-data provided by DHT. In total, six samples—AVC (dark-control), AVC (bright-control), AVC-C6 (dark-control), AVC-Ce6 (photoexcited), AVC-Pheo (dark-control), AVC-Pheo (photoexcited)—were prepared in eight replicates and were examined. Firstly, after segmentation of the representative 2D-RI maps, the binary masks indicating the regions of single cells were generated and used to count number of cells on the surface of the AVC materials 8 and 24 h after photoexcitation. The exemplary reconstructed 2D-RI maps are depicted in Figure 5. 

Obtained results demonstrate that in both dark- and bright-control samples of AVC material, an increase in the bacterial cells number occurred, which indicates that the used AVC may deliver some nutrients enabling bacterial cells reproduction. Moreover, the laser illumination did not affect the growth of the bacterial cells on the surface of this material. The reproduction rate is slightly higher for *S. aureus* than *E. coli* bacteria.

In the case of the AVC-Ce6 dark-control after 8 h of incubation, the number of bacteria cells was comparable with the number of cells of AVC without photosensitizer. However, after 24 h a decrease in the number of cells was observed for both bacteria species. As in the previous case, the division of *S. aureus* was higher than *E. coli*, which was also confirmed by the presence of the clusters of cells characteristic for biofilm formation. The analysis of photoexcited samples indicated a significant decrease in the cells’ number for both species. Obtained results confirm high (nearly 80–85% after 8 h and over 93% after 24 h) photo-inactivation efficiency of Ce6. Moreover, the results obtained for dark-control proved that photosensitizer exhibited antimicrobial properties even without photoexcitation, but the effect of the cells’ number decrease was substantially weaker.

In case of the AVC-Pheo material without the photoexcitation, the decrease of the number of cells and photo-inactivation efficiency was significantly lower than in case of the Ce6 photosensitizer, and the same tendency of lower efficiency for *S. aureus* species was observed. In the case of the photoexcited materials, the decrease of the cells’ number was observed; however, it was considerably lower than in the case of Ce6. Moreover, after 24 h the number of cells was nearly doubled in comparison with the samples after 8 h of incubation, which indicates that cells present on the material surface after 8 h were not inactivated. Obtained results show that Pheo exhibited antimicrobial properties, but its efficiency was substantially lower than in the case of Ce6. Furthermore, in the case of both dark/bright samples and for both species, cell clusters were present, indicating the possible formation of bacterial biofilm. Infrared spectroscopy confirmed that Ce6 was the more efficient source of reactive oxygen species than Pheo, which has been proven to exert antimicrobial action [58]. Moreover, the limited antimicrobial efficiency of the Pheo may be caused by its aggregation because within some regions of the reconstructed 2D-RI maps it was possible to distinguish areas of different RI values (see Figure 5B), indicating local changes of the density caused by some inhomogeneity of the material, probably associated with the presence of the Pheo aggregates. It was reported that Pheo undergoes aggregations in aqueous solution, while Ce6 remains monomeric in water [59]. Uncontrolled aggregation of photosensitizer may significantly affect photodynamic inactivation due to the weaker cells’ penetration by Pheo and inefficient photoexcitation leading to the low generation rate of free radicals. 

This observation was confirmed by obtained results of confocal microscopic examination (see Figure 4), where the significantly lower fluorescence intensity was detected for Pheo and was associated with the photosensitizer’s aggregation process. The accumulation of the PS can also be responsible for the considerably smaller changes of the averaged RI values of all bacteria cells grown on the surface of AVC-Pheo material (see Figure S3).

### 2.4. Differentiation of the Photo-Inactivated and Living Bacteria Cells by DHT

**Figure 6:**
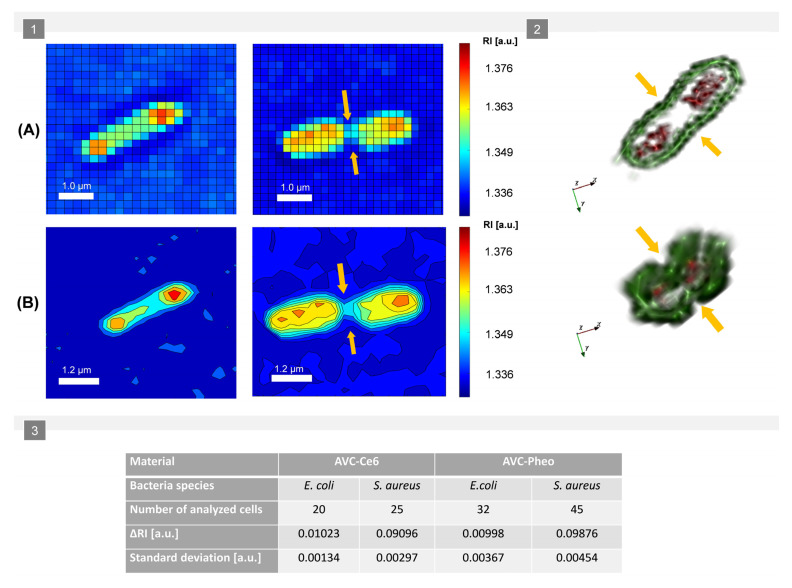
Bacteria single-cell division. (**1**) The exemplary results demonstrating the process of *E. coli* cell division by changes of its RI values: the representative average 3D-RI distribution of single-cell (**A**) and distribution of averaged 3D-RI isolines (**B**) demonstrating the process of accumulation of the cytoplasmic components on two opposite ends of the cell and the nearly complete synthetization of the septum revealed by RI data (orange arrows indicate the septum location characteristic for one of the last stages of the cell division); (**2**) the exemplary rendered and digitally stained *E. coli* and *S. aureus* dividing cells from their reconstructed 3D-RI distributions (red—corresponding to the highest RI values of cell interior, green—corresponding to the lowest RI values of the outer regions of the cell); (**3**) the changes of the averaged RI between non-dividing and dividing cells on the surface of different photoexcited materials (AVC-Ce6, AVC-Pheo).

As it was mentioned, depending on the affinity of the photosensitizer to the cellular structures, there are two possible routes of bacteria photo-inactivation by photosensitizer: disruption of the cell capsule, cell wall, or cytoplasmic membrane or inactivation of bacterial nucleoids. In the second case, the process mechanism will lead to the inactivation of the nucleoids, but the cell’s wall and membranes will maintain their continuity, and the cell with unmodified shape will still be observable on reconstructed 2D-/3D-RI maps. Therefore, to confirm that DHT can be used for differentiation of the photo-inactivated and non-inactivated cells, an additional examination, was performed. After 8 h of incubation of bacteria with photoexcited materials (AVC-Ce6, AVC-Pheo), the averaged 3D-RI of cells was determined. At that stage, the photo-inactivated cells should not have been able to divide and not have been subjected to characteristic changes and transformations related to the cell division process. According to classical microbiology, the bacterial cell prepares for division by enlarging its cell wall, cell membrane, and overall volume. Next, the septum begins to grow inward, and the chromosomes move toward opposite ends of the cell. Other cytoplasmic components of the cell are distributed to the two developing cells, leading to a local increase of the density at the opposite ends of the cell. Finally, the septum is synthesized completely through the cell center, and the cell membrane patches itself, creating two separate chambers. Therefore, the local changes in density caused by the characteristic distribution of the cytoplasmic components inside the cell should affect the averaged 3D-RI of the cell, which can be used to differentiate the inactivated and non-inactivated cells. The exemplary results of the averaged 3D-RI values obtained for *E. coli* cells are presented on Figure 6. 

However, the number of cells able to divide was limited among different analyzed samples. As in the previous case, an ANOVA analysis was performed to check whether the difference between the averaged 3D-RI values of *E. coli* and *S. aureus* cells on the surface of photoactive materials was statistically significant (see Table S2 in Supplementary Materials). The analysis determined that the *p*-value was lower than the assumed significance level (0.05), which indicates that the observed changes of the RI were statistically significant.

**Figure 7:**
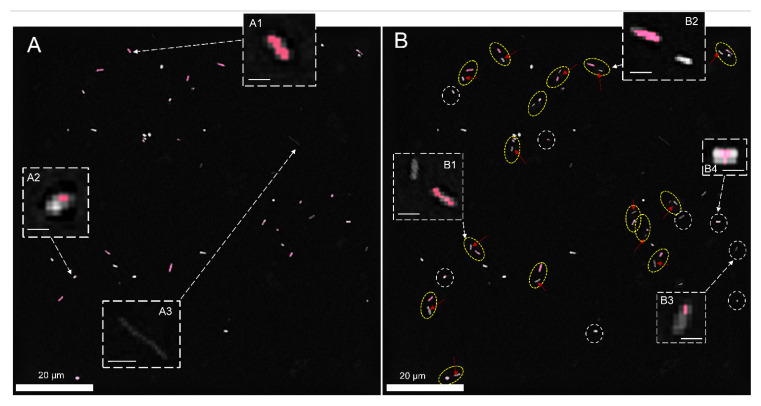
The 2D-RI maps with digitally stained cells of *E. coli:* (**A**) 8 h after photoexcitation and (**B**) 10 h after photoexcitation. Red-stained cells had RI values changing in the range from 1.368 to 1.375. The yellow ellipses indicate the stained cells, in which neighborhood the additional cells (indicated by red arrows) appear after the cell’s division. The white circles indicate initially stained cells, which turned out to be incapable of division after 2 h of the beginning of the examination. Red arrows indicate new cells not present 2 h earlier. A1–A3, B1–B3 are representative enlarged 2D-RI maps of cells (detailed description in text). The white scale bars for A1–A3, B1–B3 are equal to 2 µm.

To validate the concept of the use of DHT for differentiation of photo-inactivated cells, 8 h after photoexcitation, an additional 3 h time-lapse examination of *E. coli* cells on the surface of AVC-Ce6 material was performed. The results are shown in Figure 7. 

**Figure 8:**
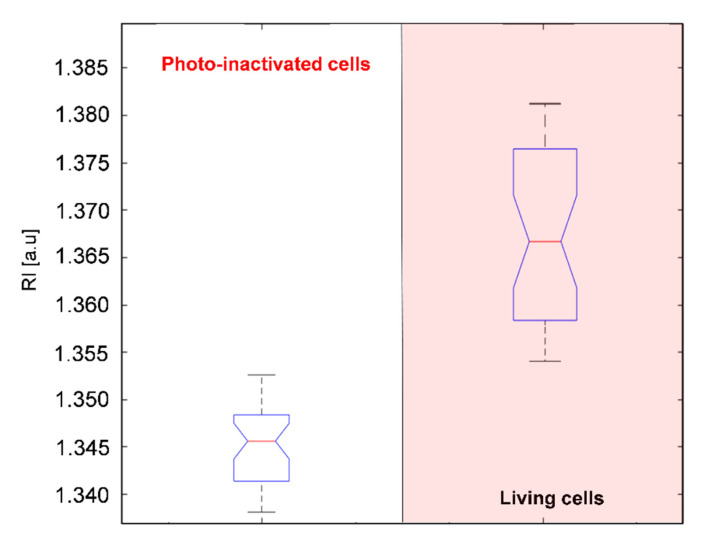
Representative boxplot showing the changes of RI values of photo-inactivated and living cells of *E. coli* on the surface of AVC-Ce6 material within 3 h of examination.

Initially, on the 2D-RI map of sample registered 8 h after photodynamic treatment, 49 cells were present. Next, the cells with RI values in the range from 1.368–1.375 were digitally stained in red color in STEVE software. This range of RI values corresponded to the reference RI values related to the living cells, as indicated in Figure 3C for *E. coli* cells on the surface of AVC-Ce6 material (dark-control). Initially, 31 cells with the RI values within in the reference range were stained (example A1 on Figure 7A), and 18 cells present on the surface of the examined material had lower RI values than 1.368 (see A3 on Figure 7A). They were also still present after 1 h. However, 8 cells initially stained after 1 h from the beginning of the examination had lower RI values than 1.3680, and no additional cells in the neighborhood, were present. In the case of these cells, the initial staining was less effective because the larger regions of these cells had lower RI values than the defined range (example A2 on Figure 7A). Out of 31 of initially stained cells, 23 cells had RI values higher than 1.3721 after 1 h, and this was related to the increase of the cytoplasm concentration characteristic of cell division. This assumption was confirmed after 2 h when in their neighborhood new cells (examples B1,B2 in Figure 7B) occurred after cell division of the initially stained cells. After 2 h, there were no additional cells (examples: B3,B4 in Figure 7B) around the 26 cells with lower RI values, apart from 18 initially stained cells and an additional 8 cells with decreased RI after 1 h. In control measurements, after 3 h most of these cells had the same low RI values, but 10 of them were not present, which suggests the death of these cells. Therefore, the decrease in RI values inside the cells (see Figure 8) may indicate the local decrease of intracellular density and changes in their chemical composition caused by the photo-induced intracellular biochemical processes accompanying the photo-inactivation. Based on the obtained results, it was possible to distinguish the ranges of RI values that corresponded to living and photo-inactivated cells. Presented results confirm that digital staining based on the RI value of single cells may be successfully used for differentiation of living and inactivated bacterial cells.

## 3. Conclusions

Quantitative phase imaging using digital holographic tomography plays an increasingly important role in biomedical examinations—particularly, as it offers significant improvements in the single cell studies by combining the advantages of other imaging modalities such as phase, confocal and fluorescence microscopies with non-destructive, label-free 3D imaging, lower phototoxicity, and no photobleaching. We demonstrated that DHT can provide significant advantages in the characterization of single-cell interaction with photosensitizers (or other active compounds). The collected results proved that the analysis of the variation of RI values of single bacterial cells can be used to characterize the accumulation of photosensitizers in a cell and to provide resources with which to observe the dynamics of the process. Moreover, the differences between the RI values can be used for digital staining and for eliminating the need of using external dyes or fluorescence markers. The benefit of the DHT method includes the visualization of the single cell’s division process based on the averaged 3D-RI distributions and differentiation of the photo-inactivated and living bacteria cells. The DHT capability for characterization of the photodynamic inactivation of bacterial cells and its penetration by the photosensitizer without photoexcitation was also confirmed. 

Digital holographic tomography was used to characterize the photodynamic inactivation efficiency of photoactive material based on ammonium acryloyldimethyltaurate copolymer with two kinds of photosensitizers. The copolymer is commonly used in pharmacology or dermatology. Therefore, its photoactive form has a potential to be used against bacterial infection in wounds, e.g., after use of orthopedic pins, external bone stabilizers, percutaneous/transdermal catheters, cannula, drainage. The patient skin barrier disruption, wounds, and close contact of these elements with tissues and blood, facilitates bacterial biofilm formation and the occurrence of bacterial infection. The use of the proposed photoactive material in the form of an antimicrobial dressing or gel can limit the possibility of bacterial infection. Obtained results demonstrate that the examined photosensitizers attach to the AVC material with a strong and stable ester bond. The use of the photoactive AVC material allows effective cell penetration by photosensitizers (particularly Ce6) and their accumulation in cells leads to over 93% photodynamic inactivation efficiency.

The performed study confirmed that DHT can be an alternative to the conventional in vitro examinations of APDT, e.g., confocal fluorescence microscopy. It enables the non-destructive characterization of the accumulation of the photosensitizer in the cell without their photoexcitation and the potentially harmful photosensitizer’s photobleaching. Therefore, the same sample may be used for further examination of the photodynamic inactivation efficiency, which significantly limits the amount of the photosensitizers used and the number of samples that need to be prepared in such an examination. Moreover, the DHT limits the typically time-consuming scanning procedure that is necessary for confocal microscopy to obtain 3D image of the samples.

Digital holographic tomography enables sensitive, objective, rapid, and reproducible measurement, and the data could be easily compared with conventional imaging such as confocal fluorescence microscopy. We demonstrated a label-free method for functional and physiological research of cellular processes in real-time without any exogenous labelling agents or dyes, such as fluorescence proteins, organic/inorganic dyes, and quantum dots. Digital holographic tomography may lead to the better understanding of photodynamic mechanisms on the single-cell level; it is particularly useful for the study of intracellular antimicrobial agent delivery, which will help development of the new generation of biomaterials that are able to combat biofilms.

## 4. Materials and Methods

The conducted study focused on the application of DHT for characterization of the changes of the bacterial single cells’ refractive index caused by the developed photoactive materials for APDT. In the first stage, the spectrophotometric and IR-spectroscopic examinations of the photoactive materials were performed. Next, the process of the accumulation or single-cell penetration by PS, was investigated by DHT and verified by fluorescence confocal microscopy on non-illuminated (dark-control samples). To examine the antibacterial efficiency of proposed materials after photoexcitation, DHT was exploited to study the photo-inactivated single cells and their reproduction ability and to determine the decrease of the number of bacteria cells, for which dedicated image processing algorithms for analysis of the series of 2D-RI maps was employed. A detailed description is presented of the materials and methods used.

### 4.1. Preparation of Photoactive Materials

As antimicrobial agents Chlorin e6 (Ce6) and Pheophorbide a (Pheo) (Santa Cruz Biotechnology Inc., Dallas, TX, USA) were selected. Their solutions were prepared in PBS (phosphate-buffered saline, ThermoFisher Scientific, Waltham, MA, USA) pH = 7.4 to obtain the maximum of the Soret band in the range 405–407 nm for luminescence measurements/microscopic observations and the strong Q-band with the maximum in the range 650–670 nm for photodynamic bacteria inactivation under laser light exposure. The photoactive materials were prepared from 20 µL of the 0.1% aq. ammonium acryloyldimethyltaurate copolymer (AVC) (Clariant International LTD., Muttenz, Switzerland) and deposited onto glass-bottom micro-dishes (µ-Dish 35 mm low, Ibidi GmbH, Gräfelfing, Germany). Photosensitizer (PS) doped materials were prepared from AVC and photosensitizer’s solutions to obtain the 17 nmol of the PSs in each sample. The area occupied by each material was equal to 30 mm^2^.

### 4.2. Spectrophotometric and Spectroscopic Characterization of Photosensitizer Materials

To characterize the photoexcitation and photodynamic properties of these materials, spectroscopic examinations were conducted by the AvaSpec-3648 spectrophotometer with 2 nm spectral resolution (Avantes Inc., Apeldoorn, The Netherlands) in the transmission mode with deuterium-halogen lamp (AvaLight-DH-S-BAL Avantes Inc., Apeldoorn, The Netherlands). The ATR-FTIR spectra of photoactive materials deposited on the slide glass were obtained using a Nicolet iN10 infrared microscope (Thermo Fisher Scientific, Waltham, MA, USA) equipped with liquid nitrogen-cooled mercury cadmium telluride (MCT-A) detector and Slide-On MicroTip Ge ATR crystal. The microscope was continuously purged with dry air. All spectra were collected in the range of 3750–675 cm^−1^ with a spectral resolution of 4 cm^−1^, averaging 128 scans. Directly before sampling, the background spectrum of germanium/air was recorded as a reference (256 scans, 4 cm^−1^). All spectra were registered at room temperature. Each sample was measured three times and then averaged to cover variation in material thickness. All spectra were analyzed using OriginPro (ver. 2019, OriginLab Corporation, Northampton, MA, USA).

### 4.3. Photoexcitation Conditions

The bright-control samples were illuminated by a laser with a wavelength of 655 nm with an adjustable power control unit coupled into the optical fiber system (FC-655 nm-1W-15070826, Changchun New Industries Optoelectronics Tech. Co., Ltd., Changchun, China). The power density was equal to 503 mW/cm^2^, and energy density was equal to 60.4 J/cm^2^. The light power measurements were performed using a highly sensitive compact fiber photodiode power sensor (S151C, Thorlabs, Newton, NJ, USA) and power meter (PM100D, Thorlabs, Newton, NJ, USA). All exposures were constantly monitored by temperature measurement with a thermal imaging camera (FLIR E6, FLIR Systems, Inc., Willsonville, OR, USA) to eliminate the possibility of cells’ overheating. The thermal imaging confirmed that there was no temperature change caused by the photoexcitation.

### 4.4. Bacterial Sample Preparation 

Two bacteria species (one Gram-positive, one Gram-negative) were examined: *Escherichia coli* (ATCC 25922) and *Staphylococcus aureus* (ATCC 25923) capable of forming biofilms. The cultures were obtained from the Department of Epizootiology and Veterinary Administration with the Clinic of Infectious Diseases of the Wroclaw University of Environmental and Life Sciences. A single colony of each species was inoculated in Brain Heart Infusion (BHI, Merck KGaA, Darmstadt, Germany) medium at 37°C. The overnight culture stocks were measured with the estimation of the MacFarland scale. The 0.5 McF was taken, which is approximately 1.5 × 10^8^ bacterial cells per ml. The bacteria were diluted 1000 times in NaCl solution; therefore, approximately 1.5 × 10^5^ bacterial cells were applied to the dish for attachment. The diluted bacterial suspension was then aliquoted using 1 mL per well of µ-dish, where the analyzed photoactive material was placed. The biofilm formation began with the irreversible cell attachment to the surface [60,61]. Therefore, to examine only cells attached to the surface of prepared photoactive materials after 2 h of incubation at 37 °C, the supernatant with planktonic cells was removed, and the well was washed twice with 2 mL of NaCl (0.9%), followed by the addition of 2 mL of NaCl (0.9%). Such procedure of the preparation of biofilm samples for characterization of different antimicrobial coating or agents was already reported [62,63]. The refractive index of the NaCl (0.9%) was equal to 1.335 and was measured by the Abbe refractometer (NAR-2T, minimum scale: 0.001, ATAGO Co. Ltd., Tokyo, Japan) at 20 °C. For further examination, the wells with the same number of bacterial cells were chosen. Next, the samples were incubated under the same conditions for the next 4 h. After that, the eight samples of each material (AVC, AVC-Ce6, AVC-Pheo) were irradiated for 2 min (bright-control/treated-photoexcited samples), and eight samples of the same materials were not irradiated and used as dark-control. Then, all samples were incubated at 37 °C for the next 24 h. After 8 and 24 h of incubation, the samples were examined by DHT.

### 4.5. Digital Holographic System and Measurements Conditions

**Figure 9:**
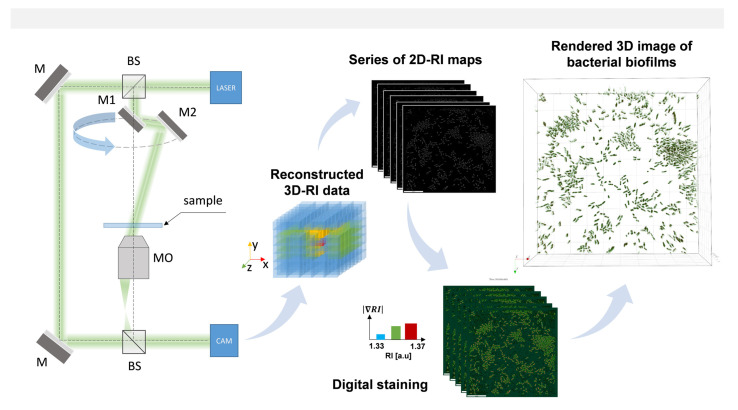
The schema of the experimental system and the RI data processing. M, flat mirrors; BS, beam splitters; MO, microscopic objective; M1,M2, mirrors on the rotational arm; CAM, digital camera.

Measurements by means of DHT were performed to characterize the 3D-RI data distribution of single cells on AVC materials (without/with photosensitizers) and to differentiate the non-treated, treated (by photosensitizers), and photo-inactivated single cells based on the variation of the spatial distribution and values of their RI. The projections needed for acquiring the 3D-RI maps were registered as individual digital holograms (DHs) of a sample under illumination from different angles by scanning an object illuminating beam while the camera and the sample remained at the fixed locations. The DHs resulting from the superposition of the reference plane wave and object wave scattered by samples were registered using commercial off-axis Mach–Zehnder interferometric setup (see Figure 9) with a rotatable scanning mirror (3D Cell Explorer, Nanolive, Ecublens, Switzerland). The series of DHs were registered at the wavelength 520 nm (sample exposure 0.2 mW mm^−2^) by a dry microscope objective (60×, numerical aperture NA = 0.8, Nikon, Tokyo, Japan) for each position of the scanning mirror. The phase and amplitude of the scattered waves were retrieved from DHs and then processed for the reconstruction of the 3D-RI map and their rendered 3D visualizations by utilizing the STEVE software (version 1.6.3496, Nanolive, Ecublens, Switzerland). Additionally, it was possible to digitally stain bacterial cells based on their spatial distribution of RI values, enabling label-free characterization. In total, six samples—AVC (dark-control), AVC (bright-control), AVC-C6 (dark-control), AVC-Ce6 (photoexcited), AVC-Pheo (dark control), AVC-Pheo (photoexcited)—were prepared in eight replicates and were examined. For each from the 48 samples, over 30 3D-RI maps were registered. Each 3D-RI map contained 95 slices (2D-RI tomograms) reconstructed with the axial resolution 367 nm.

### 4.6. Qualitative and Quantitative Analysis of the Single Cells’ RI Data

**Figure 10:**
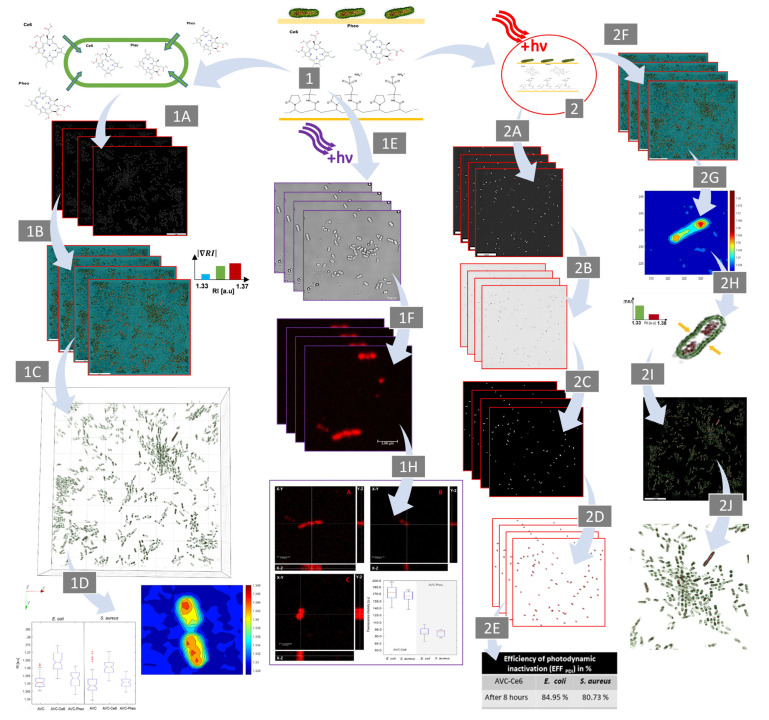
Schema of the bacteria samples’ examination. Single cells treated and untreated by PS (**1**), as well as cells in the presence of PS after photodynamic effect induction (**2**). Detailed explanation of the steps followed is described in text.

DHT was used to characterize the changes in RI values of bacterial cells treated and untreated by photosensitizers and to examine the influence of single cell penetration by PS on its RI (see Figure 10(1A,1B)). After registration of the series of DHs, it was possible to retrieve the 2D-RI map (2D-RI tomogram) from each DH (see Figure 10(1A)) and, based on them, to obtain the 3D-RI distributions of single cells. 

These distributions were further used to determine the RI variation and digital staining of bacterial cells (see Figure 10(1B)), which enabled the rendered 3D visualization of bacterial cells (see Figure 10(1C)) and quantitative analysis (see Figure 10(1D)). To analyze the 3D-RI of bacterial cells, it was necessary to examine a series of 2D-RI maps (slices) obtained from the registered 3D-RI distributions. Next, each slice had to be processed using the global segmentation algorithm based on cluster method [47] to obtain areas occupied by single cells. Next, the averaged 3D-RI values of the pixels corresponding to the region in which the single cells were present on all slices were determined. In our approach, the RI variation of the PS-treated cells in comparison with PS-untreated cells indicate the effectiveness of the cell’s penetration by PS and efficacy of photodynamic inactivation. To determine the existence of statistically significant RI variation of cells caused by the PS accumulation, a one-way ANOVA (Analysis of Variance) was performed, with a significance level equal to 0.05 and based on the assumption that all RI-data populations were normally distributed. The normality assumption of the extracted average 3D-RI values of single cells was verified by the Anderson–Darling test. All image processing, as well as the determination of the average 3D-RI values and statistical analysis, were performed using MATLAB^®^ software (version R2020a, MathWorks, Natick, MA, USA).

### 4.7. Confocal Microscopic Imaging of Bacterial Cells

A scanning confocal microscope (Leica TCS SPE, Leica, Wetzlar, Germany) was used to visualize the penetration of the bacterial cells by photosensitizer and to verify the results obtained by DHT (see Figure 10(1E–1H)). This microscope was working in fluorescence and differential interference contrast (DIC) modes. The microscope was equipped with standard lasers in fluorescence mode (405 nm, 488 nm, 532 nm, and 635 nm) and a halogen lamp used in DIC mode. The microscopic imaging parameters such as the type and wavelength of the laser excitation light, the diameter of the pinhole, and the laser intensity were controlled automatically by the microscope’s software (Leica Application Suite, LAS V.3.1, Cambridge Ltd., Cambridge, UK). Observation of the photosensitizer’s luminescence was carried out using the high-pass filter with cutting wavelength at 575 nm. The samples of bacterial cells grown on the AVC materials (AVC-Ce6, AVC-Pheo) were imaged by the confocal microscope (oil immersion objective 63×, NA = 1.3, Leica) working in DIC mode (see Figure 10(1E)). Next, the samples were photoexcited by the laser with a wavelength of 405 nm (see Figure 10(1F)) to generate the PS’s luminescence signal. The DIC and fluorescence images were combined, and the X-Z, Y-Z cross-sections (see Figure 10(1H)) were retrieved to obtain 3D information, to identify which cells’ regions were spatially overlapping the fluorescence, and to indicate the PS’s presence and its localization. For characterization of the efficiency of the PS accumulation inside the single cells, the mean fluorescence signals’ intensity in the region of single cells was determined.

### 4.8. Analysis of the Photodynamic Inactivation of Bacteria Cells

The photo-inactivation of bacterial cells by photosensitizer can lead to the disruption of the cell’s wall and membranes or inactivation of bacterial nucleoids, depending on the affinity of the PS to given cellular structures [64,65,66,67]. The first mechanism leads to the overflow of cytoplasm into the external environment. In consequence, destroyed cells will be not observed on 2D/3D-RI maps. The second mechanism will lead to inactivation of the nucleoids, but the morphological structures will not be affected as quickly; thus, they will maintain their continuity and will be observed on reconstructed 2D-/3D-RI maps. To take into account these two mechanisms, a two-stage examination was proposed.

The first-stage analysis (see Figure 10(2F–2J)) was focused on the comparison of the averaged 3D-RI of single cells after photoexcitation with the cells from bright-control that were still able to reproduce. Digital holographic tomography time-lapse examination of the samples 2 h and 8 h after photoexcitation was performed. To demonstrate the possible decrease of the bacterial cells’ growth and biofilm formation by photodynamic inactivation induced by the two kinds of PSs, three kinds of materials were examined: AVC, AVC-Ce6, and AVC-Pheo. For each material, two kinds of samples were used: illuminated by the light of 655 nm (bright-control/excited) and not illuminated (dark-control). The digitally stained 2D-RI maps (see Figure 10(2F)) were used for the characterization of the variation of the cross-sectional 2D distribution of RI inside the cell (see Figure 10(2G)), as well as the 3D distributions (see Figure 10(2H)), which enabled the differentiation of living and photo-inactivated cells (see Figure 10(2I–2J)). Again, one-way ANOVA was performed to determine whether the observed variations of the averaged 3D-RI values of single cells from different samples were statistically significant. 

The second-stage analysis (see Figure 10(2A–2E)) was focused on the counting of single cells on the surface of the examined materials. The reconstructed 2D-RI maps at appropriate depth with the largest cells’ cross-section (see Figure 10(2A)) were determined. After initial contrast reversion (see Figure 10(2B)) and application of the segmentation algorithm, the determination of the region occupied by cells and acquisition of the binary mask were conducted (see Figure 10(2C)). For automatic counting of bacterial cells, the freeware ImageJ Analyze Particles plugin [68] was used (see Figure 10(2D)). After determination of the number of living cells, it was possible to determine the efficiency of the photodynamic effect for analyzed photoactive materials (see Figure 10(2E)). Typically, crystal violet is used to determine the bacteria concentration in biofilm and to evaluate cells attachment to the surface. However, this technique was unsuitable in our examination because of the absorption of crystal violet by AVC. Therefore, the determination of antimicrobial efficiency was performed based on the direct analysis of the RI data obtained by DHT and the counting of bacterial cells. The analysis of the efficacy of photodynamic inactivation (*EFF_PDI_*) was performed on over 30 3D-RI maps for 12 samples: 8 (Equation (1)) and 24 h (Equation (2)) after photoexcitation using the following math formulas:

(1) $$EFF_{PDI}^{8h}=\frac{N_{dark\;AVC+PS}^{8h}-N_{excited\;AVC+PS}^{8h}}{N_{dark\;AVC+PS}^{8h}}$$

(2) $$EFF_{PDI}^{24h}=\frac{N_{dark\;AVC+PS}^{24h}-N_{excited\;AVC+PS}^{24h}}{N_{dark\;AVC+PS}^{24h}}$$

where $N_{dark\;AVC+PS}^{8h}$ is the mean value of the number of bacteria cells in dark-control samples after 8 h, $N_{excited\;AVC+PS}^{8h}$ is the mean value of the number of bacteria cells in samples 8 h after PS excitation by laser irradiation (λ = 655 nm) for 2 min, $N_{dark\;AVC+PS}^{24h}$ is the mean value of the number of bacteria cells in dark-controls samples after 24 h, and $N_{excited\;AVC+PS}^{24h}$ is the mean value of the number of bacteria cells in samples 24 h after PS excitation by laser irradiation (λ = 655 nm) for 2 min. 

## Supplementary Materials

The following are available online at https://www.mdpi.com/article/10.3390/ijms22105068/s1, **Figure S1.** (A) The representative ATR-FTIR spectra of photosensitizers before (black line) and after laser irradiation (red and green line, respectively) in range of 3600–2800 cm^−1^, corresponding to stretching vibrations of OH, NH, and CH groups (left) and in range of 1800–1500 cm^−1^ dominated by *n*(C = O) and δ(NH) bands (right); (B) The representative ATR-FTIR spectra of AVC material (black line), AVC-Ce6 (green line), and AVC-Pheo (red line) photosensitizers with (C) zoomed spectra in the range of 1800–1500 cm^−1^; **Figure S2**. The ATR-FTIR spectra of Aristoflex™ AVC before (black line) and after laser irradiation (grey line); **Figure S3.** The boxplot represents the variation of the averaged RI of *E. coli* and *S. aureus* cells depending on the material used: AVC, AVC-Ce6, AVC-Pheo. The central mark indicates the median, and the bottom and top edges of the box indicate the 25th and 75th percentiles. The whiskers extend to the most extreme data points not considered outliers, and the outliers are plotted using the ‘+’ symbol.; **Table S1**. The results of the ANOVA, where each of 6 groups represent the set of average RI of *E. coli* and *S. aureus* cells for each material: AVC, AVC+Ce6, AVC+Pheo (dark-controls); **Table S2**. The results of the ANOVA of the averaged RI of set of 20 dividing and non-dividing *E. coli* and *S. aureus* cells on the surface of AVC+Ce6, AVC+Pheo materials (bright-controls).
