# Supplementary material for: Bacteria Single-Cell and Photosensitizer Interaction Revealed by Quantitative Phase Imaging
Source: Int J Mol Sci. 2021 May 11;22(10):5068. doi: 10.3390/ijms22105068 (PMC8151141; doi:10.3390/ijms22105068)
Supplement: Supplementary file 1 [file ijms-22-05068-s001.zip › ijms-1193566-supplementary.pdf]

International Journal of *Molecular Sciences*

MDPI

Supplementary Materials

Bacteria single-cell and photosensitizer interaction revealed by quantitative phase imaging

Igor Buzalewicz[1]*, Agnieszka Ulatowska-Jarża[1], Aleksandra Kaczorowska[1], Marlena Gąsior-Głogowska[1], Halina Podbielska[1], Magdalena Karwańska[2], Anna K. Matczuk[3], Alina Wieliczko[2], Marta Kopaczyńska[1]

1 Department of Biomedical Engineering, Faculty of Fundamental Problems of Technology, Wrocław University of Science and Technology, 27 Wybrzeże S. Wyspiańskiego St. Wrocław 50-370, Poland; agnieszka.ulatowska-jarza@pwr.edu.pl (A.U.-J.); aleksandra.kaczorowska@pwr.edu.pl (A.K.); marlena.gasior-glogowska@pwr.edu.pl (M.G.-G.); halina.podbielska@pwr.edu.pl (H.P.); marta.kopczynska@pwr.edu.pl (M.I.K);

2 Department of Epizootiology and Veterinary Administration with Clinic of Infectious Diseases, Wrocław University of Environmental and Life Sciences, 45 Grunwaldzki Square Wrocław 50-366, Poland; magdalena.karwanska@upwr.edu.pl (M.K.); alina.wieliczko@upwr.edu.pl (A.W.);

3 Department of Pathology, Division of Microbiology, Faculty of Veterinary Medicine Wrocław University of Environmental and Life Sciences, 31 C.K. Norwida St. 51-375 Wrocław, Poland; anna.matczuk@upwr.edu.pl (A.M.);

* Correspondence: igor.buzalewicz@pwr.edu.pl

Four pages of suplementary materials including two supplementary figures (Fig. S1, Fig.S2,) and two supplementary tables (Table S1, Table S2).

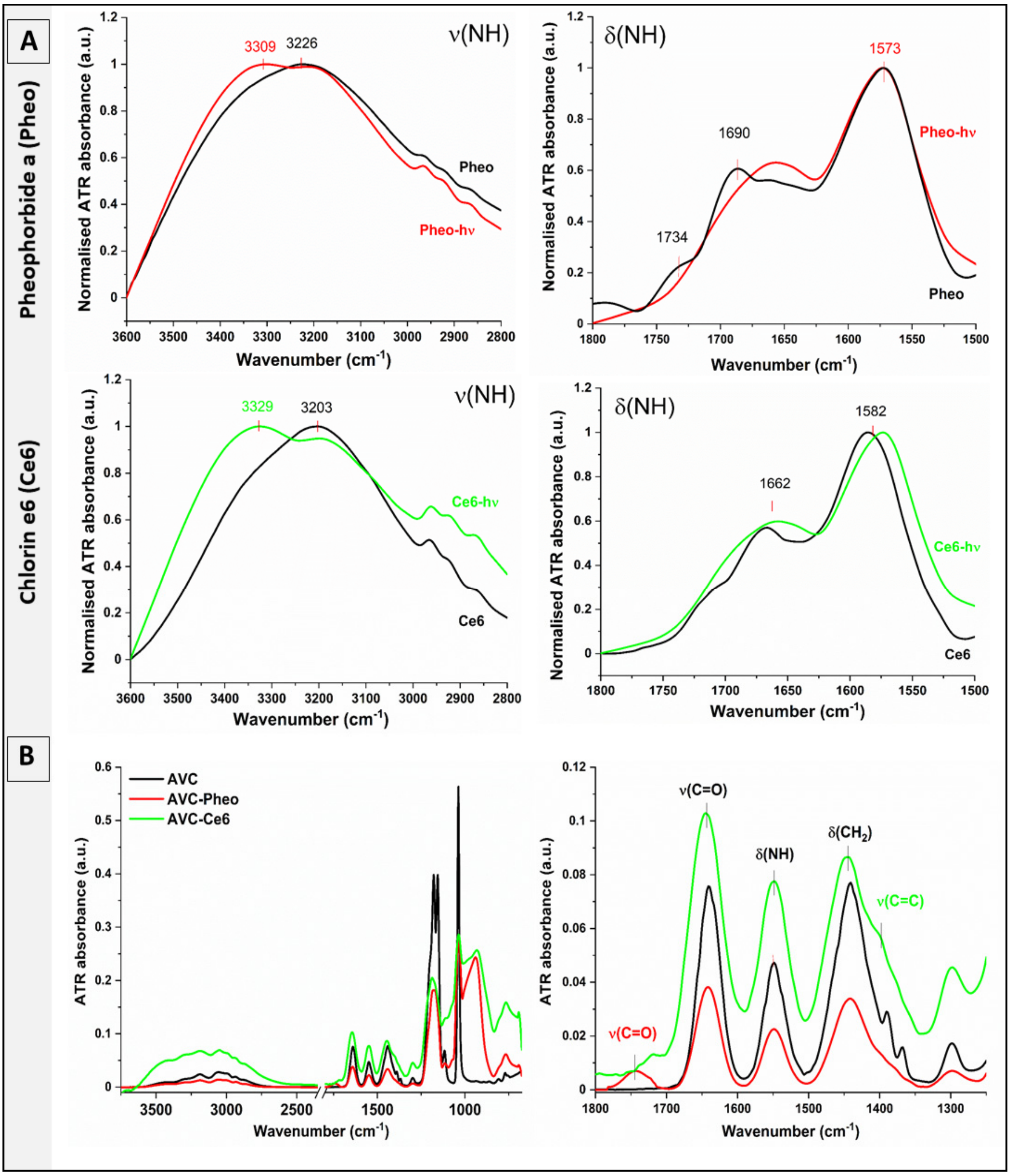

Figure S1. (A) The representative ATR-FTIR spectra of photosensitizers before (black line) and after laser irradiation (red and green line, respectively) in range of 3600-2800 cm^{-1} corresponding to stretching vibrations of OH, NH and CH groups (left) and in range of 1800-1500 cm^{-1} dominated by n(C=O) and δ(NH) bands (right); (B) The representative ATR-FTIR spectra of AVC-material (black line), AVC-Ce6 (green line) and AVC-Pheo (red line) photosensitizers with (C) zoomed spectra in the range of 1800-1500 cm^{-1}.

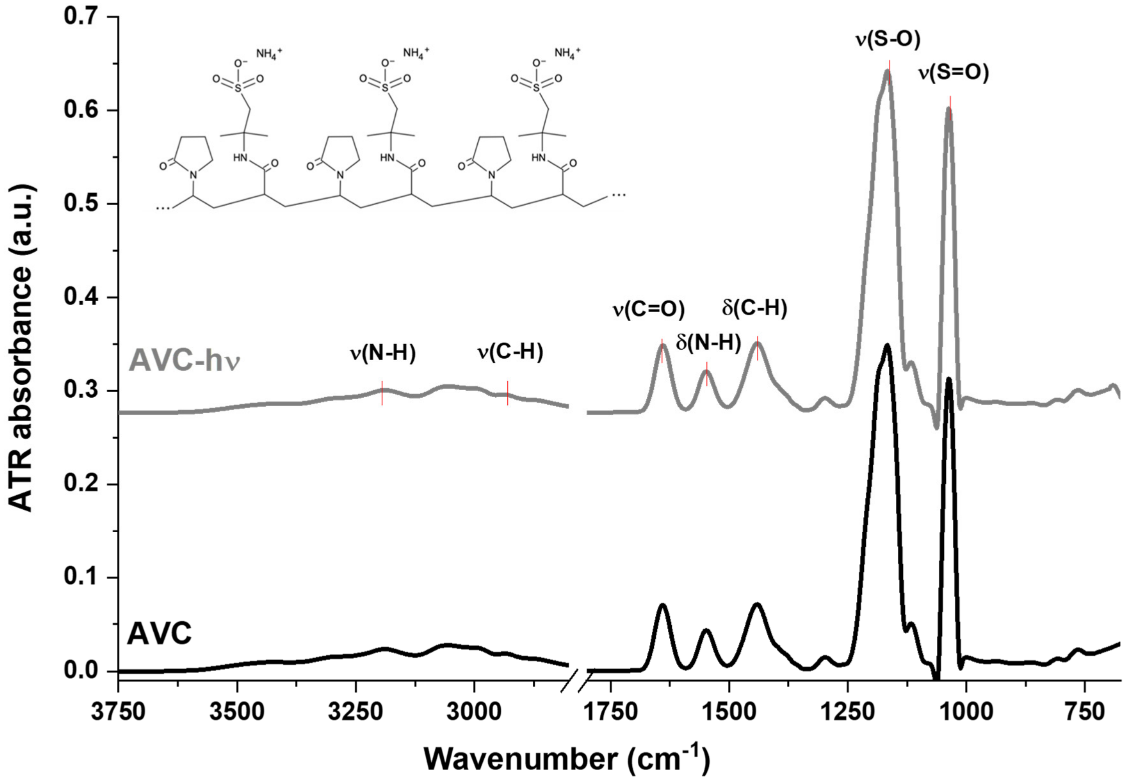

Figure S2. The ATR-FTIR spectra of Aristoflex™ AVC before (black line) and after laser irradiation (grey line).

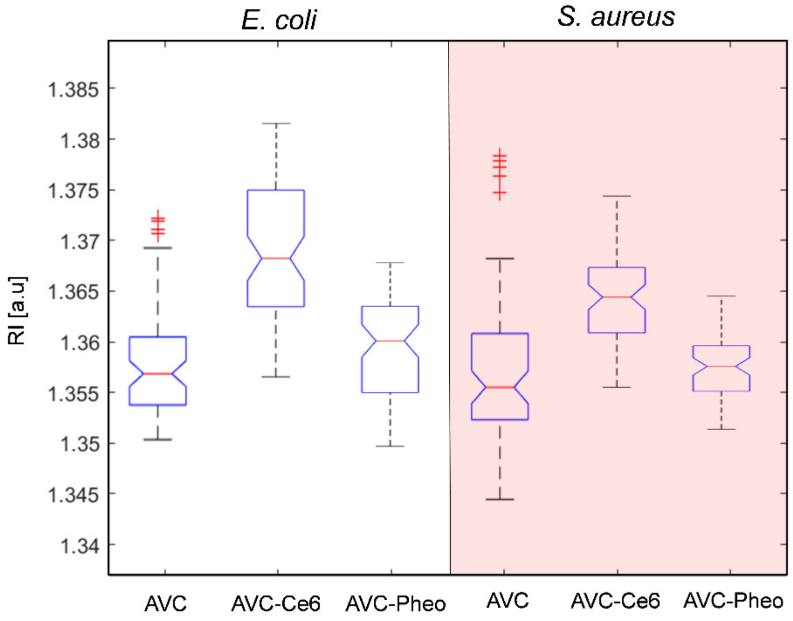

Figure S3. The boxplot represents the variation of the averaged RI of *E. coli* and *S. aureus* cells depending on the used material: AVC, AVC-Ce6, AVC-Pheo. The central mark indicates the median, and the bottom and top edges of the box indicate the 25th and 75th percentiles. The whiskers extend to the most extreme data points not considered outliers, and the outliers are plotted using the '+' symbol.

Table S1. The results of the ANOVA, where each from 6 groups represent the set of average RI of *E. coli* and *S. aureus* cells for each material: AVC, AVC+Ce6, AVC+Pheo (dark-controls).

**Source of variability**	**SS [1]**	**df [1]**	**MS[1]**	**F [1]**	**Prob>F**
Group (between)	0.01467	5	0.00293	38.03	6.159×10^{-32}
Error (within)	0.03102	1673	0.00008		
Total	0.04569	1673			

[1] SS – is a sum of squares due to each source, df – degree of freedom associated with each source, MS – mean squares for each source, F – F [1] SS – is a sum of squares due to each source, df – degree of freedom associated with each source, MS – mean squares for each source, F – F statistics, Prob>F – p-value which is the probability that F-statistic can take a value larger than computed F-statistic value.

Table S2. The results of the ANOVA of the averaged RI of set of 20 dividing and non-dividing *E. coli* and *S. aureus cells* on the surface of AVC+Ce6, AVC+Pheo materials (bright-controls).

Source of variability	SS [1]	df [1]	MS[1]	F [1]	Prob>F
Group (between)	0.00122	5	0.0061	12.01	2.13316×10^{-5}
Error (within)	0.00502	114	0.00005		
Total	0.00624	120			

[1] SS – is a sum of squares due to each source, df – degree of freedom associated with each source, MS – mean squares for each source, F – F statistics, Prob>F – p-value which is the probability that F-statistic can take a value larger than computed F-statistic value.
